# Artemisinin Attenuates Transplant Rejection by Inhibiting Multiple Lymphocytes and Prolongs Cardiac Allograft Survival

**DOI:** 10.3389/fimmu.2021.634368

**Published:** 2021-02-24

**Authors:** Zhe Yang, Fei Han, Tao Liao, Haofeng Zheng, Zihuan Luo, Maolin Ma, Jiannan He, Lei Li, Yongrong Ye, Rui Zhang, Zhengyu Huang, Yannan Zhang, Qiquan Sun

**Affiliations:** Research Institute of Organ Transplantation, The Third Affiliated Hospital of Sun Yat-sen University, Guangzhou, China

**Keywords:** artemisinin, cardiac transplantation, transplant rejection, T cell-mediated rejection, antibody-mediated rejection

## Abstract

Immunological rejection is an important factor resulting in allograft dysfunction, and more valid therapeutic methods need to be explored to improve allograft outcomes. Many researches have indicated that artemisinin and its derivative exhibits immunosuppressive functions, apart from serving as a traditional anti-malarial drug. In this assay, we further explored the therapeutic effects of artemisinin for transplant rejection in a rat cardiac transplantation model. We found that it markedly attenuated allograft rejection and histological injury and significantly prolonged the survival of allograft. Upon further exploring the mechanism, we demonstrated that artemisinin not only attenuated T cell-mediated rejection (TCMR) by reducing effector T cell infiltration and inflammatory cytokine secretion and increasing regulatory T cell infiltration and immunoregulatory cytokine levels, but also attenuated antibody-mediated rejection (ABMR) through inhibition of B cells activation and antibody production. Furthermore, artemisinin also reduced macrophage infiltration in allografts, which was determined to be important for TCMR and ABMR. Moreover, we demonstrated that artemisinin significantly inhibited the function of pure T cells, B cells, and macrophages *in vitro*. All in all, this study provide evidence that artemisinin significantly attenuates TCMR and ABMR by targeting multiple effectors. Therefore, this agent might have potential for use in clinical settings to protect against transplant rejection.

## Introduction

Cardiac transplantation has become the gold-standard long-term medical treatment for end-stage heart failure and has achieved remarkable success (1). However, its biggest challenge is concomitant rejection, resulting from interactions between the recipient immune system and allograft. Immune responses could result in the rejection to cardiac allografts, which including chronic rejection (CR) and acute rejection (AR). And AR is a leading cause to the development of CR (2). Furthermore, transplant rejection mainly consists of T cell-mediated rejection (TCMR) and antibody-mediated rejection (ABMR) (3). Although immunosuppression has achieved good results for TCMR therapy, side effects such as infection and graft toxicities are likely to be a significant impact on patient prognosis (4). Moreover, ABMR, caused by donor-specific antibodies (DSAs), has emerged as an important immunological barrier to successful transplantation (5, 6). Current therapeutic strategies for ABMR focus on removing the generated antibodies in the peripheral blood, or eliminating B cells to inhibit the generation of antibodies, but these strategies are not as effective as expected (7). Therefore, more effective and safe strategies must be explored to improve transplant outcomes.

Artemisinin (C_15_H_22_O_5_, ART), derived from sweet wormwood (Artemisia annua L), has been used to treat malaria in China for a long time. Taking the high safety without noticeable side effects and adverse reactions into consideration, ART has been used to treat millions patients in the world (8). Recently, ART and its derivatives were found to have other properties, including immunosuppressive and anti-inflammatory effects (9).

ART and its derivatives exhibit immunosuppressive and against inflammation *via* regulation of the immune system, inhibiting T lymphocyte proliferation, enhancing Treg differentiation, and increasing the proliferation of Treg (10–12). Dihydroartemisinin (derivative of ART) treatment also decreases autoantibodies in lupus model (13). However, as ART could obviously inhibit the activation and proliferation of T cells, the decrease of antibody level was considered as a secondary effect on the inhibition of T cells. and the direct effects of these drugs on B cells (especially for DSAs) are unclear. Moreover, ART can to regulate innate immune cells. Studies have indicated that ART significantly reduces peritoneal macrophage phagocytosis and the phagocytic index *in vivo* and can also inhibit monocyte/macrophage adhesion to HUVECs to protect against the development of early atherosclerotic lesions (14, 15). To date, the anti-inflammatory and immunosuppressive properties of ART have been discussed, but their effect on immunological rejection has not been reported.

Therefore, here, we investigated the therapeutic effects of ART on rejection, including TCMR and ABMR, using a rat cardiac transplantation model, providing a novel therapy choice for the patients undergoing transplant rejection.

## Materials and Methods

### Reagents

ART (purity: 99.83%, Selleck Chemicals, California, USA) was dissolved in DMSO at 100 mM for storage and use. Antibodies used for immunohistochemical staining included anti-CD3 (ab16669,1:800), anti-CD8 (ab217344,1:400), anti-CD68 (ab31630, 1:1600), anti-Foxp3 (ab22510, 1:400; all from Abcam, Cambridge, England), anti-CD4 (Cell Signaling Technology, Shanghai, China; D7D2Z, 1:800), and anti-rat C4d (Hycult Biotech, Uden, Netherlands; HP8034, 1:400). Anti-rat antibodies for flow cytometry were from BioLegend, including CD45-Pacific Blue, CD3-FITC, CD4-APC/Cy7, CD8a-PerCP, and CD11b- PE/Cy7. B220-PE was purchased from Thermo Fisher.

### Experimental Protocol and Groups

The model of cardiac allograft transplant rejection was established by transplanting Brown Norway (BN) hearts into Lewis recipient rats. Recipients were random assigned to two groups as follows: (a) ART (n=6), ART was diluted in peanut oil (100 g/L) and administered intragastrically daily at 22 mg/kg/day from day 0 until cardiac graft rejection; (b) control (n=6), an equal volume of peanut oil was applied to vehicle-treated recipients. Cardiac Lewis-to-Lewis rat transplantation served as the isograft control (n=5), and these animals all received a daily equal volume of peanut oil administered intragastrically. For rats only undergoing skin transplantation, animals were divided into two groups as follows: (1) ART, ART was fed intragastrically (22 mg/kg/day) from day 0 to day 36 post skin transplantation; (2) control, an equal volume of peanut oil was applied to control group animals every day.

### Subjects and Animals

Peripheral blood was obtained from healthy volunteers between the ages of 18 and 26 years who were acquired from Sun Yat-sen University. All subjects signed informed consent forms and our study was approved by the Ethical Committee of Sun Yat-sen University.

Male rats weighing 200–250 g were obtained from Vital River Laboratory Animal Technology Co., Ltd. Animals used in this assay were proved by the Sun Yat-sen University Institutional Ethical Guidelines and consistent with the Guide for the Care and Use of Laboratory Animals.

### Rat Skin Transplantation

For skin transplantation, the donor rat was anesthetized with isoflurane, the tail was excised, and full-thickness tail skin was obtained. The recipient was anesthetized, and full-thickness skin grafts (1–2.0 × 2 cm^2^) were acquired from BN donors. The grafts were then transplanted onto the dorsal area of Lewis rats with an 8–0 nylon suture.

### Rat Heterotopic Cardiac Transplantation

Detailed processes of cardiac transplantation were described previously (16, 17). Briefly, the pulmonary artery and ascending aorta of the heart were anastomosed end-to-end to the vena cava and abdominal aorta, respectively. Survival of cardiac graft function was judged everyday by abdominal palpation. Rejection was defined as total cessation of contractions.

### Detection of Circulating Donor-Specific Antibodies

Graft recipient sera were obtained at the indicated time points. Circulating DSA (IgM and IgG) was evaluated using flow cytometry. Briefly, donor splenocytes were incubated with recipient sera for 30 min at 37°C, washed, and then incubated with anti-rat IgM and IgG (Bio Legend) for 1 h at 4°C. The mean fluorescence intensity was used to reflect individual DSAs.

### Histology and Immunohistochemistry

Five days post cardiac transplantation, cardiac grafts were fixed and embedded. Hematoxylin and eosin (H&E), anti-CD3, anti-CD4, anti-CD8, anti-CD68, and anti-Foxp3 were used for further assessment. Sections were examined by light microscopy to evaluate the pathologic features of cardiac allografts. For TUNEL staining, the one-step TUNEL apoptosis assay kit (Roche TMR-RED; 12156792910) was used and strict limited to the protocol. Sections were counterstained with DAPI to label nuclei. Fields containing both the rejection area and the normal zone were scanned.

### Flow Cytometry

Fresh recipient cardiac grafts or spleens were digested in RPMI 1640 with collagenase 1 and DNAse for 60 min at 37°C. Thereafter, cells were stained using antibodies for CD45, CD11b, B220, CD3, CD8a, and CD4, results were analyzed using a CytoFLEX flow cytometer (Beckman Coulter).

### Quantitative Real-Time Polymerase Chain Reaction

mRNA levels were determined using RT-qPCR. RNA was acquired from fresh tissues or cells using TRIzol reagent (Invitrogen, USA). PrimeScript RT master mix (TAKARA, Japan) was used for reversing transcribed. SYBR Green I master mix (Roche, Switzerland) was used for RT-qPCR in a LightCycler480 system (Roche), with *GAPDH* as an internal control. Detailed Primers used could be found in **Table 1**.

**Table 1:** List of primers used for qPCR.

Gene	Forward primer	Reverse primer
***CD3***	TTCAAGATAGAAGTGGTTGAATATG	CACCTCCTTCGCCAGCTCC
***CD4***	TGTGTCAGGTGCCGGCACCAACAG	GTGGGGCCCAGGCCTCATATG
***CD8***	AGGGAATGGGATTGGGCTTCGC	CTCTGAAGGTCTGGGCTTGAC
***CD19***	CACCCATGGTTCATTGCCCA	GCACAACATTGTCTCCCTCTTC
***Foxp3***	TGCAGCTCCGGCAACTTTTC	TGTCTGAGGCAGGCTGGATA
***IL-1β***	CAGCTTTCGACAGTGAGGAGA	TTGTCGAGATGCTGCTGTGA
***IL-2***	GCAGGCCACAGAATTGAAAC	CCAGCGTCTTCCAAGTGAA
***IL-6***	CTTCCAGCCAGTTGCCTTCT	GACAGCATTGGAAGTTGGGG
***IL-10***	TGCTATGTTGCCTGCTCTTACTG	TCAAATGCTCCTTGATTTCTGG
***IL-17***	GGAGAATTCCATCCATGTGCC	GGCGTTTGGACACACTGAAC
***IFN-γ***	ATCCATGAGTGCTACACGCC	TCGTGTTACCGTCCTTTTGC
***TGF-β***	TTGCTTCAGCTCCACAGAGA	TGGTTGTAGAGGGCAAGGAC
***GAPDH***	CATCAACGACCCCTTCATTGA	ACTCCACGACATACTCAGCACC

### Isolation and *In Vitro* Culture of T Cells, B Cells, and Macrophages

Corresponding microbeads (Miltenyi Biotec, Germany) were used for cells isolation from peripheral blood mononuclear cells (PBMCs). Flow cytometry was used for determining purity of the cells, and the purity was consistently >95%.

In the presence or absence of ART, purified T-cells (CD3^+^ cells) were incubated for 48 h with anti-CD3 plus anti-CD28, purified B-cells (CD19^+^ cells) were incubated for 9 days with anti-CD40L plus anti-IgM, and purified macrophages (CD14^+^ cells) were incubated for 48 h with LPS (lipopolysaccharide). PBMCs and isolated cells were cultured in RPMI-1640 (100 U/ml penicillin,10% FBS, and 100 mg/ml streptomycin) at 37°C with 5% CO_2_.

### Assessment of Apoptosis

An annexin V apoptosis detection kit (R&D Systems) was used for apoptosis detection according to the manufacturer’s instructions using flow cytometry (BD, San Diego, CA).

### Enzyme-Linked Immunosorbent Assay

Commercially available ELISA kits used to measure IFN-γ, IL-1β, or Ig levels in *in vitro* culture system supernatants as follows: IFN-γ, IL-1β, IgG, and IgM ELISA kits from Thermo Fisher (San Diego, CA, USA, catalog:88-7316-88, 88-7261-88, 88-50550, and 88-50620).

### Statistical Analysis

The KS normality test was first used to test if the data were normally distributed; all data met this criterion. The Student’s t tests were used for analysis of significant differences. The log-rank test was used for graft survival. Data are expressed as the mean ± standard deviation (SD). Statistical analyses were performed using Prism (GraphPad). *P < 0.05 was considered significant.

## Results

### ART Reduces Rejection and Prolongs Cardiac Allograft Survival

**Figure 1:**
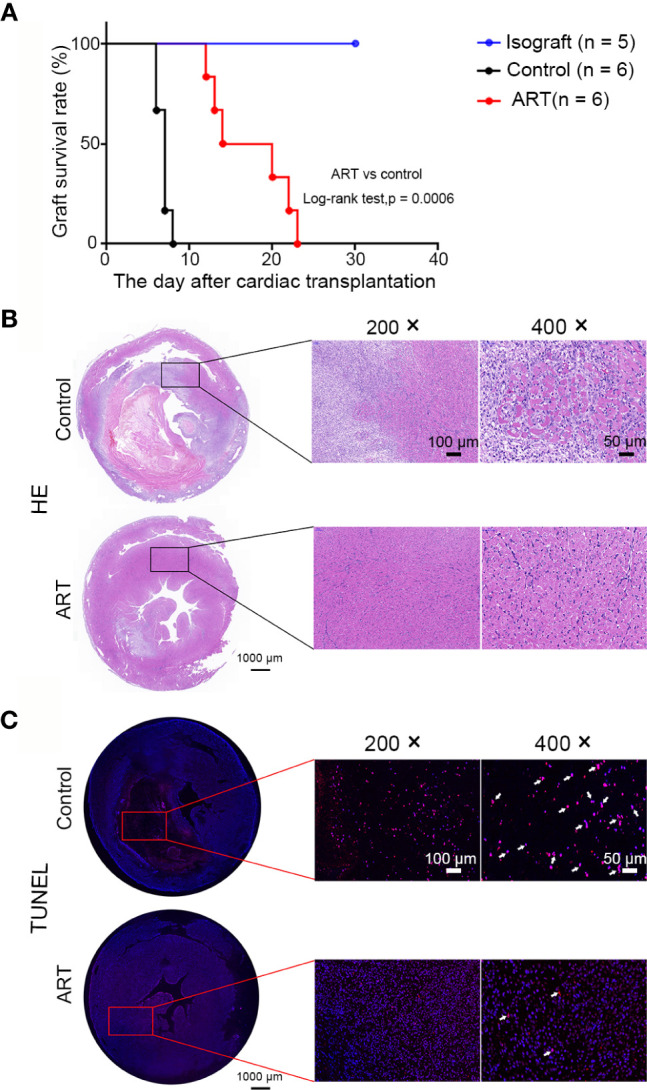
Artemisinin (ART) reduces inflammatory inflltration, myocyte damage, and apoptosis of cells to prolong cardiac allograft survival. **(A)** ART significantly prolonged allograft survival compared to that in controls. **(B)** Histologic evaluation of cardiac allografts post-transplant. Hematoxylin and eosin staining showing interstitial vasculitis, hemorrhage, edema, and myocyte damage. **(C)** Representative images of heart sections showing TUNEL-positive cells (red, arrowheads indicated) in the rejection area; nuclei are stained with DAPI (blue). TUNEL, (TdT)-mediated nick end-labeling; DAPI, 4’,6-diamidino-2-phenylindole.

Recipients treated with ART had longer graft survival than controls (17.33 ± 4.89 vs 6.83 ± 0.75 days); approximately 66% of grafts in the ART group survived over 2 weeks, while survival time of grafts were less than 8 day in the control group (**Figure 1A**). Subsequently, allografts were harvested on day 5 after cardiac transplantation. As shown by representative H&E photomicrographs, the rejection area of the ART group was noticeably thinner than that of the control group. Interstitial vasculitis, hemorrhage, and edema were evident in the control group, which were significantly diminished by ART treatment (**Figure 1B**). Moreover, TUNEL staining revealed apoptotic cells (red) in the ART group were significantly less than that in the control group, especially in the rejection area. DAPI staining (blue) indicated that nuclei in the control group were deformed and broken, which indicated that more cells were in a state of apoptosis. Together, these data indicate that ART reduced rejection and protected cardiomyocytes (**Figure 1C**).

### ART Attenuates T Cell-Mediated Rejection, and Increasing Regulatory T Cell Infiltration in Allografts

**Figure 2:**
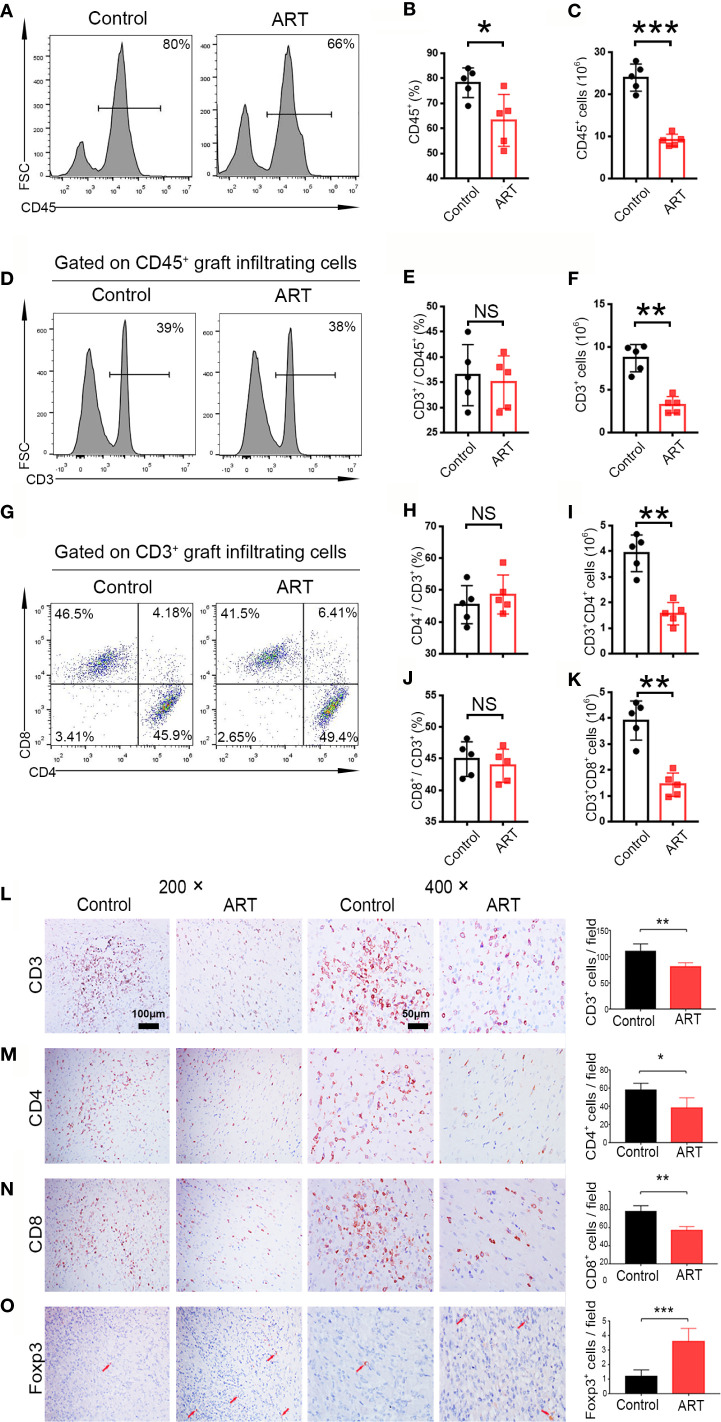
Artemisinin (ART) reduces T cell infiltration in cardiac allografts. Lymphocytes (CD45^+^), T cells (CD3^+^), CD4^+^ T cells (CD3^+^CD4^+^), and CD8^+^ T cells (CD3^+^CD8^+^) in cardiac allografts were detected by flow cytometry. Representative histograms and quantitative analysis of frequencies and counts of lymphocyte cells **(A–C)**, T cells **(D–F)**, CD4^+^ T cells and CD8^+^ T cells in allografts **(G–K)** (n = 5/group). **(L–O)** Representative immunohistochemistry staining images and results of quantitative analysis of cell numbers/view based on CD3, CD4, CD8, and Foxp3 in the Control and ART groups (n = 5/group). Magnification: 200× and 400×. *P < 0.05; ** *P* < 0.01; *** *P* < 0.001. NS, no significance.

**Figure 3:**
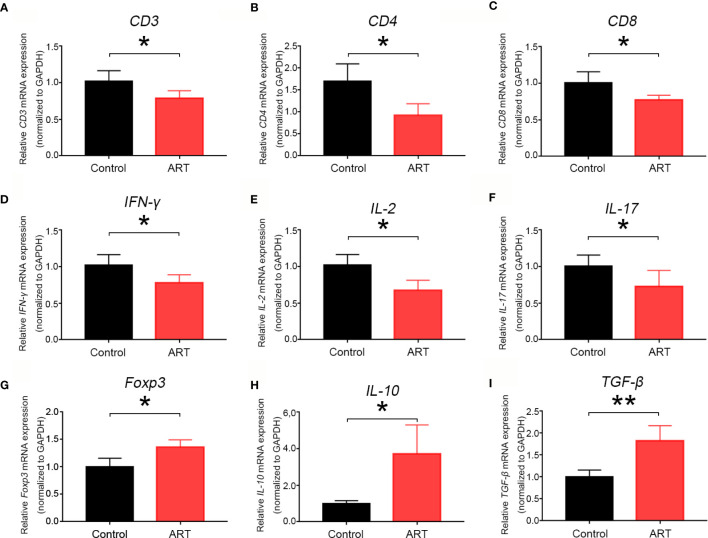
Artemisinin (ART) inhibits mRNA expression levels of T cell-associated markers and pro-inflammatory cytokines and promotes mRNA expression of Treg- associated cytokines. The mRNA expression of T cell-associated markers **(A–C)**, T-cell-associated pro-inflammatory cytokines **(D–F)**, and Treg-associated cytokines **(G–I)** were determined by qPCR. The mRNA levels were normalized to those of *GAPDH*. Three independent experiments were performed showing similar results. * *P* < 0.05; ** *P* < 0.01.

Five days after transplantation, inflammatory cell (CD45^+^) frequencies and numbers were significantly lower in the cardiac grafts obtained from the ART group compared with the control group (**Figures 2A–C**). As shown in **Figures 2D–K**, infiltrative CD3^+^, CD3^+^ CD4^+^, and CD3^+^ CD8^+^ cell numbers were significantly less in cardiac grafts of the ART group than in those of the control group, although the percentages of these cells did not change significantly. As shown by representative IHC stains, ART treatment significantly decreased the infiltration of T cells (**Figures 2L–N**), and the results showed the same trend as flow cytometry results. Intriguingly, ART treatment also dramatically enhanced Treg cell (Foxp3^+^) expansion based on IHC results (**Figure 2O**). Additionally, mRNA levels of the T cell-associated genes *IL-2*, *IFN-γ*, *CD3*, *CD8*, *CD4*, and *IL-17* were significantly reduced following ART treatment (**Figures 3A–F**). Furthermore, those of Treg cell-associated genes, *Foxp3*, *IL-10*, and *TGF-β*, were significantly elevated (**Figures 3G–I**).

### ART Inhibits B Cells Activation and Donor-Specific Antibodies Production and Attenuates Allograft Antibody-Mediated Rejection

**Figure 4:**
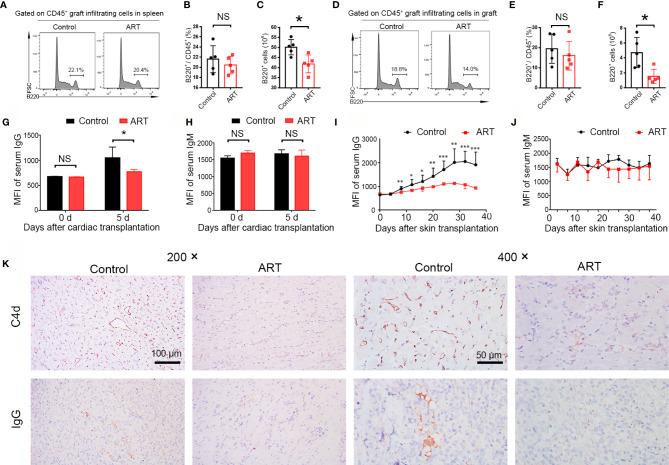
Artemisinin (ART) attenuates antibody-mediated rejection *via* the inhibition of B cell and donor specific antibody (DSA) production. **(A–F)** B cells (B220^+^) were detected by flow cytometry in fresh spleens and allografts on day 5 after cardiac transplantation. Representative histograms showing the frequencies and counts of B cells in host spleens **(A–C)** and cardiac grafts **(D–F)** are presented. **(G, H)** Changes in DSA levels (IgG and IgM) on day 0 and 5 after cardiac transplantation (n = 5). **(I, J)** Changes in DSA levels (IgG and IgM) after Lewis rats that received skin allografts from BN donors were treated with (ART group) or without ART (control group) for 36 days. **(K)** Representative immunohistochemistry stained images showing the deposition of C4d and IgG. Magnification: 200× and 400×. * *P* < 0.05; ** *P* < 0.01; *** *P* < 0.001. NS, no significance.

ABMR, caused by B cell activation and DSA production, is an important factor in allograft dysfunction. By flow cytometry, we examined B cell (B220^+^) populations in spleens and allografts of recipients obtained 5 days after transplantation and quantitatively analyzed frequencies and cell numbers. Although there was no effect on B cell frequencies, ART treatment decreased the number of B cells in the spleens and cardiac grafts compared with the control group (**Figures 4A–F**). Furthermore, changes in DSA levels (IgG and IgM) at 0 and 5 days after cardiac transplantation were detected; ART treatment conspicuously inhibited this upward trend (**Figure 4G**). No significantly difference was found regarding the levels of IgM between the ART -treated and control groups (**Figure 4H**). To further explore the influence of ART on DSAs, we established a rat skin transplant model. Levels of IgG increased at day 8 post skin transplantation gradually, whereas ART significantly inhibited the upward trend, with 16.1%, 22.3%, 22.8%, 30.8%, 35.5%, 43.9%, 47.7%, and 51.2% reductions at days 8, 12, 16, 20, 24, 28, 32, and 36, respectively (**Figure 4I**). On the contrary, limited response was found regrading IgM levels post skin allograft transplantation (**Figure 4J**). Moreover, ART treatment significantly reduces diffuse C4d and IgG deposition in capillaries (**Figure 4K**).

### ART Reduces Macrophage Infiltration Into Allografts

**Figure 5:**
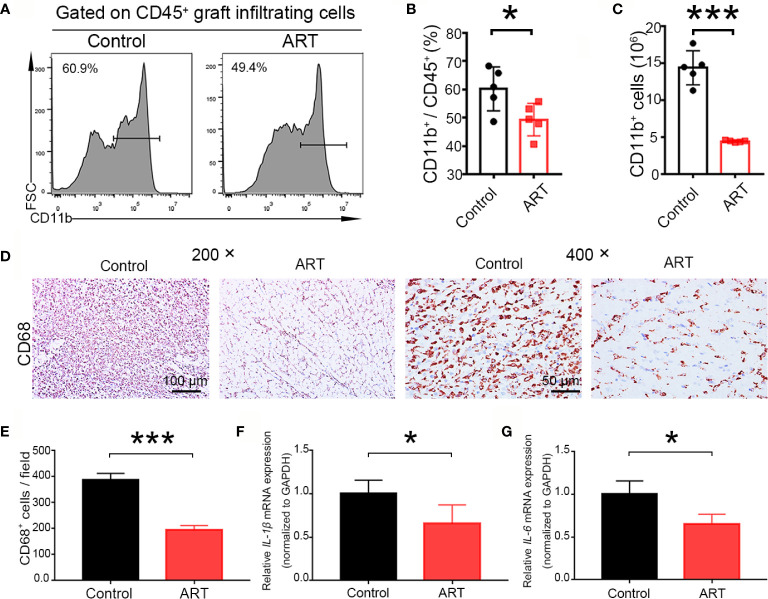
Artemisinin (ART) suppresses the number and function of macrophages *in vivo*. **(A–C)** Macrophages (CD11b^+^) were detected by flow cytometry in allografts on day 5 after cardiac transplantation, and the representative histograms for frequencies and counts of macrophages in cardiac grafts **(D, E)** are shown. Representative immunohistochemistry staining images and quantitative analysis of cell number/view, based on CD68, in the control and ART groups (n = 5/group). **(F, G)** ART inhibited the mRNA expression of *IL-1β* and *IL-6*. The mRNA levels were normalized to those of *GAPDH*. Three independent experiments were performed showing similar results. Data were expressed as the mean ± SD. * *P* < 0.05; *** *P* < 0.001. Magnification: 200× and 400×.

According to the diagnosis of heart rejection, interstitial mononuclear and macrophage cell infiltration is the typical feature of AR, and macrophage is of vital importance in the development of TCMR and ABMR (18, 19). Therefore, macrophage infiltration in the allografts were detected. Less frequencies of macrophages (CD11b^+^) were found in the ART -treated group than the control group (49.34 ± 5.77% vs. 60.16 ± 7.76%, *P* < 0.05), and macrophage numbers were reduced more clearly (**Figures 5A–C**). Representative histological analysis also revealed ART treatment inhibited infiltration of CD68 cells (**Figures 5D, E**). Moreover, levels of pro-inflammatory cytokines (*IL-6* and*IL-1β*) were decreased in the ART group when compared with the control group, indicating that macrophage function was inhibited by ART treatment (**Figures 5F, G**).

### Artemisinin Significantly Suppresses T Cell, B Cell, and Macrophage Function *In Vitro*

**Figure 6:**
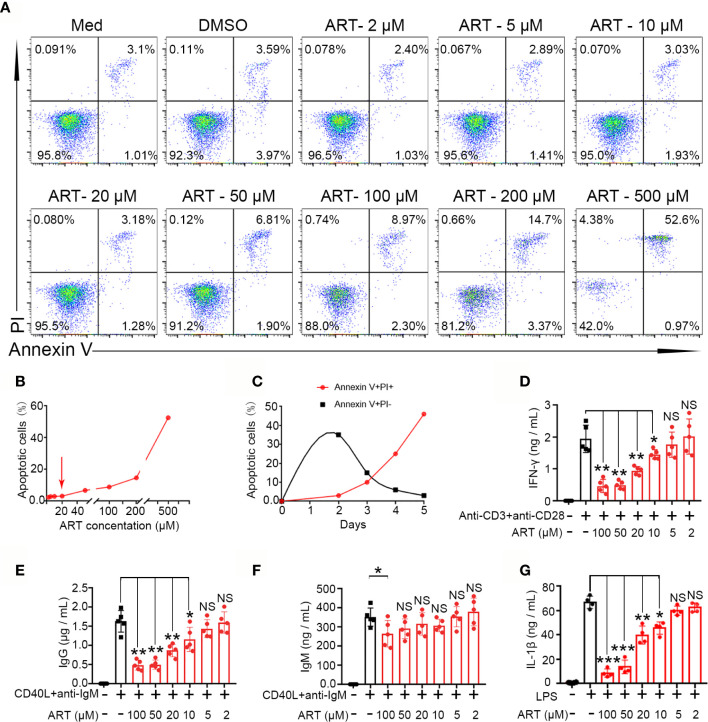
Artemisinin (ART) inhibits T cell, B cell, and macrophage differentiation and reduces cytokines secretion *in vitro*. **(A, B)** Effect of ART on cell apoptosis was assessed by PI staining. **(C)** The dynamics of cell death induced by ART (20 µM) were measured by PI and annexin V staining. **(D)** Purified CD3^+^ T cells from PBMCs were stimulated with or without anti-CD3 plus anti-CD28 (1 µg/ml) in the presence or absence of various concentrations (2 to 100 μM) of ART. The cell culture supernatants were collected at 48 h and the level of IFN-γ was measured by ELISA (n = 5). **(E, F)** Purified CD19^+^ cells from PBMCs were stimulated with or without CD40L (1 μg/ml) plus anti-IgM (5 μg/ml) in the presence or absence of various concentrations (2 to 100 μM) of ART. The cell culture supernatants were collected at 9 days and the levels of IgG and IgM were measured by ELISA (n = 5). **(G)** Purified CD14^+^ T cells from PBMCs were stimulated with or without LPS (1 μg/ml) in the presence or absence of various concentrations (1 to 20 μM) of ART. The cell culture supernatants were collected at 48 h and the levels of IL-1β were measured by ELISA (n = 5). Statistical results are shown as the mean ± SD. *P < 0.05; **P < 0.01, ***P < 0.001. PI, propidium iodide; PBMC, peripheral blood mononuclear cell; ELISA, enzyme linked immunosorbent assay. NS, no significance.

To further explore mechanisms underlying the suppression of lymphocyte function by ART, we performed *in vitro* assays using PBMCs. Because ART can induce apoptosis, to exclude the potential confounding effects of reduced cytokine secretion, which might result from apoptosis, we independently assessed the proapoptotic effect of ART on total lymphocytes. Specifically, PBMCs were cultured with ART at concentrations ranging from 2 to 500 µM for 24 h, and PI-Annexin V staining was performed to evaluate cell survival. ART promoted apoptosis in a dose-dependent manner (**Figure 6A**). Finally, our results showed that at 20 µM, the pro-apoptotic effect of ART was mild, while apoptosis occurred when cells were cultured over 20 µM ART (**Figure 6B**). Moreover, measurements of ART (20 µM)-induced cell death overtime revealed the rapid accumulation of annexin V^+^PI^−^ cells at the early stage of culture, which transitioned to annexin V^+^PI^+^ cells over time (**Figure 6C**). Therefore, subsequent *in vitro* experiments were performed with a range of ART centered around 20 µM.

Next, we purified specify cells, including B cells (CD19^+^), T cells (CD3^+^), and monocytes (CD14^+^) from human peripheral mononuclear leukocytes and cultured them with different ART concentrations to evaluate cytokine secretion *in vitro*. As shown in **Figure 6D**, purified CD3^+^ T cells from PBMCs produced high levels of IFN-γ following stimulation of anti-CD3 plus anti-CD28. Further, cytokine productions were inhibited by ART in a dose-dependent manner. A significant inhibitory effect was observed at 10 µM, and a decrease in cytokines was induced at higher concentrations (~100 µM). B cells (CD19^+^) purified from PBMCs were stimulated with CD40L and anti-IgM for 9 days with or without ART at different concentrations and supernatant IgG and IgM levels were evaluated by ELISA. ART also inhibited IgG production in a dose-dependent manner at 10 µM. Except for with 100 µM ART, IgM levels were not significantly changed (**Figures 6E, F**). As mentioned, purified CD14^+^ cells produced high levels of IL-1β following LPS stimulation. However, ART, from 10 to 100 µM, significantly inhibited this in a dose-dependent manner (**Figure 6G**).

## Discussion

ART and its derivatives are associated with potent immunosuppressive function and have been involved in some clinical trials dealing with autoimmune diseases (20, 21). Here, we provide the first evidence demonstrating the effect of ART on transplant immunity using a rat cardiac transplantation model. Specifically, it significantly inhibited rejection by targeting multiple effectors, attenuated allograft injury, and thus prolonged cardiac allograft survival.

Currently, transplant rejection is recognized as a dangerous factor for allograft loss (22). Thus, sustainable treatment of rejection remains critical for long-term graft function. Although immunosuppressive therapy has dramatically prolonged the lives of patients, the notable problems associated with these drugs remain. First of all, significant toxicities which affect allograft survival should be taken into consideration. Second, their long-term use inevitably increases infection risks and it might lead to death, especially in the first year post-transplantation (23). Therefore, safe and effective immunosuppression in clinical practice is expected.

Fully MHC-mismatched cardiac transplantation was used to establish a rat transplant rejection model. Rejection diagnosis and classification were based on histological changes, which occur with allograft injury, and inflammatory cell infiltration, and experiments have indicated that rejection is a mix of humoral and cellular rejection (24). In this assay, macrophages and T cells were the major subsets of immune cells infiltrating into the grafts, in accordance with previous results (25). We also observed B cell infiltration and C4d and IgG deposition in the allografts, suggesting that ABMR was involved in rejection in this model. Further, ART significantly attenuated TCMR and ABMR by inhibiting T cell, B cell, and macrophage activation and infiltration. More importantly, approximately 66% of recipients administered ART survived beyond 14 days without combinations with any other drugs or strategies, this is unprecedented.

T cell is of vital importance in rejection post allogeneic transplantation. Recognize allo-antigens by T cell is the primary basis for allograft rejection (23). ART and its derivative were shown to inhibit T lymphocytes. Wang et al. demonstrated that artemether (an ART derivative) has direct inhibitory effects on T cells, suppressing T cell proliferation and activation *via* the Ras–Raf1–ERK1/2 (11). Another study found that SM934 (an ART derivative) inhibits the accumulation and differentiation of Th1 and Th17 cells and induces the differentiation and expansion of Treg cells (26). In our study, all cardiac grafts were rejected within 8 days post transplantation when no other interventions were applied. Flow cytometric and histological analysis showed that ART treatment significantly inhibited infiltration of lymphocytes, especially effector T cells, in allografts and reduced inflammatory cytokine secretion. Moreover, functions of pure T cells were inhibited by ART *in vitro* in a dose-dependent manner. These results indicated that ART could significantly inhibit the occurrence of TCMR. Intriguingly, ART treatment also dramatically enhanced the expansion of Treg cells, which play a central role in transplant tolerance. This is consistent with other assays and our previously published data showing that Treg cell therapy could significantly alleviate renal allograft injury and induce immune tolerance in animal and clinical trials (27, 28). Our data thus showed that ART could effectively inhibit TCMR and induce Treg cell expansion.

ABMR occurs when recipients are presensitized to donor antigens before surgery or due to *de novo* DSA production post-operatively. B cells are the main effectors of ABMR. To explore the changes in B cells, we first observed that ART decreased these cell numbers in recipient spleens and allografts. Although function of infiltration B cell in grafts is a mystery, Hippen et al. demonstrated that B cells might be involved in the deterioration of allograft function and anti-donor antibody production (29). These data provided evidence that infiltration of B cell as a critical parameter of ABMR. Therefore, ART treatment could reduce B cell infiltration in the recipient spleen and allografts.

Although some studies have demonstrated that ART family drugs reduce antibodies in a model of systemic lupus erythematosus, it was not known if ART could decrease DSAs with organ transplantation (13, 30). We generated a new animal model of BN skin transplantation into Lewis recipients to detect changes in DSAs in the circulation of different treatment groups. This study, for the first time, demonstrated that ART dramatically reduces circulating DSA-IgG levels in this model. Moreover, the changes in DSA-IgG levels at 0 and 5 days after cardiac transplantation were similar. Histological changes, including IgG and C4d were decreased in ART-treated group. Current therapeutic strategies to control DSAs are focused on antibody removal with plasma exchange, intravenous immunoglobulin, and monoclonal antibodies (31, 32). However, these strategies also have some limitations, although a combination of multiple strategies could contribute to a synergistic effect (33, 34). Owing to its excellent suppressive effects on production of DSAs, ART might be an economical and effective choice for therapy of AMR in clinical settings.

Apart from adaptive immune cells, other innate immune cells including macrophages, monocytes, and NK cells infiltrate allografts during rejection and were proven to aggravate allograft injuries (18, 35, 36). Moreover, macrophages are important innate immune cells; in human cardiac transplants, the infiltrates associated with rejection are predominantly composed of mononuclear cells and macrophages with macrophages outnumbering T cells, especially in the prophase of rejection (37, 38). Moreover, recent studies have found that innate myeloid immune cells, such as macrophages and monocytes, can form antigen-specific immune memory and thus serve as an central component in immune rejection (39). All of these results indicate that macrophage is of vital importance during transplantation immune response. Accordingly, our results showed that macrophages were outnumbered T cells, and ART treatment could significantly reduce the frequency and number of macrophages compared to those in the control group. Pro-inflammatory cytokine *IL-1β* were also decreased in the ART group. These results also proved that ART treatment can not only inhibit the number of infiltrating macrophages but also reduce their function.

Although our results indicated that ART could significantly attenuate rejection by inhibiting multiple pathways, several limitations should be noted. First, effects of ART are wide, and we did not conduct studies on such mechanisms in depth. Second, the research on immune cell function was not thorough, and especially, the subtype analysis of cytokines was not sufficient. Finally, the concentration of ART *in vivo* was determined based on our experience and references, and thus, the side effects of ART should be further explored.

In summary, we established a transplant rejection model and demonstrated that ART can suppress TCMR, ABMR, and macrophage infiltration *in vivo* and provided evidence that ART attenuates allograft injury and rejection after rat cardiac transplantation. Subsequently, we demonstrated the inhibitory effect of ART on the function of various purified lymphocytes *in vitro*. Although this was a pilot study of the suppressive effects of ART on rejection, this study provides novel evidence for the therapy of rejection in patients who suffer transplantation.

## Data Availability Statement

The original contributions presented in the study are included in the article/supplementary material. Further inquiries can be directed to the corresponding authors.

## Ethics Statement

The animal study was reviewed and approved by Sun Yat-sen University Institutional Ethical Guidelines for animal experiments.

## Author Contributions

ZY: study design and drafting of the manuscript. FH: performed the model. TL: performed the model and helped write the manuscript. HZ: performed the experiments. ZL: performed the experiments. MM: performed the transplantation model. JH: performed the transplantation model. LL: generated the immune- histochemistry data. YY: labeled the image and statistical analysis. RZ: labeled the image and statistical analysis. ZH: critical revision of the manuscript. YZ: conceived the study and performed the experiments. QS: conceived the study and critical revision of the manuscript. All authors contributed to the article and approved the submitted version.

## Funding

This study was supported by the National Natural Science Foundation of China (No. 81970649, 81970650, 81770753, 81800662, and 81800663); the National Key R&D Program of China (2018YFA0108804); Guangdong Basic and Applied Basic Research Foundation (2019A1515011942); and the PhD Start-up Fund of Natural Science Foundation of Guangdong Province, China (2018A030310324); the China Postdoctoral Science Foundation (2020M683083).

## Conflict of Interest

The authors declare that the research was conducted in the absence of any commercial or financial relationships that could be construed as a potential conflict of interest.

## References

[1] Dhital KK Iyer A Connellan M Chew HC Gao L Doyle A. Adult heart transplantation with distant procurement and ex-vivo preservation of donor hearts after circulatory death: a case series. Lancet (2015) 385(9987):2585–91. 10.1016/S0140-6736(15)60038-1 25888085

[2] Meier-Kriesche HU Ojo AO Hanson JA Cibrik DM Punch JD Leichtman AB. Increased impact of acute rejection on chronic allograft failure in recent era. Transplantation (2000) 70(7):1098–100. 10.1097/00007890-200010150-00018 11045649

[3] Bhagra SK Pettit S Parameshwar J. Cardiac transplantation: indications, eligibility and current outcomes. Heart (2019) 105(3):252–60. 10.1136/heartjnl-2018-313103 30209127

[4] Lund LH Edwards LB Kucheryavaya AY Benden C Christie JD Dipchand AI. The registry of the International Society for Heart and Lung Transplantation: thirty-first official adult heart transplant report–2014; focus theme: retransplantation. J Heart Lung Transpl (2014) 33(10):996–1008. 10.1016/j.healun.2014.08.003 25242124

[5] Lefaucheur C Loupy A Vernerey D Duong-Van-Huyen JP Suberbielle C Anglicheau D. Antibody-mediated vascular rejection of kidney allografts: a population-based study. Lancet (2013) 381(9863):313–9. 10.1016/S0140-6736(12)61265-3 23182298

[6] Loupy A Toquet C Rouvier P Beuscart T Bories MC Varnous S. Late Failing Heart Allografts: Pathology of Cardiac Allograft Vasculopathy and Association With Antibody-Mediated Rejection. Am J Transpl (2016) 16(1):111–20. 10.1111/ajt.13529 26588356

[7] Colvin MM Cook JL Chang P Francis G Hsu DT Kiernan MS. Antibody-mediated rejection in cardiac transplantation: emerging knowledge in diagnosis and management: a scientific statement from the American Heart Association. Circulation (2015) 131(18):1608–39. 10.1161/CIR.0000000000000093 25838326

[8] Klayman DL. Qinghaosu (artemisinin): an antimalarial drug from China. Science (1985) 228(4703):1049–55. 10.1126/science.3887571 3887571

[9] Ho WE Peh HY Chan TK Wong WS. Artemisinins: pharmacological actions beyond anti-malarial. Pharmacol Ther (2014) 142(1):126–39. 10.1016/j.pharmthera.2013.12.001 24316259

[10] Li T Chen H Yang Z Liu XG Zhang LM Wang H. Evaluation of the immunosuppressive activity of artesunate in vitro and in vivo. Int Immunopharmacol (2013) 16(2):306–12. 10.1016/j.intimp.2013.03.011 23583335

[11] Wang JX Tang W Shi LP Wan J Zhou R Ni J. Investigation of the immunosuppressive activity of artemether on T-cell activation and proliferation. Br J Pharmacol (2007) 150(5):652–61. 10.1038/sj.bjp.0707137 PMC2189761 17262016

[12] Zhao YG Wang Y Guo Z Gu AD Dan HC Baldwin AS. Dihydroartemisinin ameliorates inflammatory disease by its reciprocal effects on Th and regulatory T cell function via modulating the mammalian target of rapamycin pathway. J Immunol (2012) 189(9):4417–25. 10.4049/jimmunol.1200919 PMC3478428 22993204

[13] Dong YJ Li WD Tu YY. [Effect of dihydro-qinghaosu on auto-antibody production, TNF alpha secretion and pathologic change of lupus nephritis in BXSB mice]. Zhongguo Zhong Xi Yi Jie He Za Zhi (2003) 23(9):676–9. 14571616

[14] Wang Y Cao J Fan Y Xie Y Xu Z Yin Z. Artemisinin inhibits monocyte adhesion to HUVECs through the NF-kappaB and MAPK pathways in vitro. Int J Mol Med (2016) 37(6):1567–75. 10.3892/ijmm.2016.2579 PMC4866958 27122190

[15] Yang Z Ding J Yang C Gao Y Li X Chen X. Immunomodulatory and anti-inflammatory properties of artesunate in experimental colitis. Curr Med Chem (2012) 19(26):4541–51. 10.2174/092986712803251575 22834815

[16] Liao T Li Q Zhang Y Yang Z Huang Z Han F. Precise treatment of acute antibody-mediated cardiac allograft rejection in rats using C4d-targeted microbubbles loaded with nitric oxide. J Heart Lung Transplant (2020) 39(5):481–90. 10.1016/j.healun.2020.02.002 32115364

[17] Liao T Liu X Ren J Zhang H Zheng H Li X. Noninvasive and quantitative measurement of C4d deposition for the diagnosis of antibody-mediated cardiac allograft rejection. EBioMedicine (2018) 37:236–45. 10.1016/j.ebiom.2018.10.061 PMC6286270 30385231

[18] Ko JH Lee HJ Jeong HJ Kim MK Wee WR Yoon SO. Mesenchymal stem/stromal cells precondition lung monocytes/macrophages to produce tolerance against allo- and autoimmunity in the eye. Proc Natl Acad Sci U S A (2016) 113(1):158–63. 10.1073/pnas.1522905113 PMC4711840 26699483

[19] Stewart S Winters GL Fishbein MC Tazelaar HD Kobashigawa J Abrams J. Revision of the 1990 working formulation for the standardization of nomenclature in the diagnosis of heart rejection. J Heart Lung Transpl (2005) 24(11):1710–20. 10.1016/j.healun.2005.03.019 16297770

[20] Jin O Zhang H Gu Z Zhao S Xu T Zhou K. A pilot study of the therapeutic efficacy and mechanism of artesunate in the MRL/lpr murine model of systemic lupus erythematosus. Cell Mol Immunol (2009) 6(6):461–7. 10.1038/cmi.2009.58 PMC4003040 20003822

[21] Li Y Wang S Wang Y Zhou C Chen G Shen W. Inhibitory effect of the antimalarial agent artesunate on collagen-induced arthritis in rats through nuclear factor kappa B and mitogen-activated protein kinase signaling pathway. Transl Res (2013) 161(2):89–98. 10.1016/j.trsl.2012.06.001 22749778

[22] Lund LH Khush KK Cherikh WS Goldfarb S Kucheryavaya AY Levvey BJ. The Registry of the International Society for Heart and Lung Transplantation: Thirty-fourth Adult Heart Transplantation Report-2017; Focus Theme: Allograft ischemic time. J Heart Lung Transpl (2017) 36(10):1037–46. 10.1016/j.healun.2017.07.019 28779893

[23] Lechler RI Sykes M Thomson AW Turka LA. Organ transplantation–how much of the promise has been realized? Nat Med (2005) 11(6):605–13. 10.1038/nm1251 15937473

[24] Kfoury AG Miller DV Snow GL Afshar K Stehlik J Drakos SG. Mixed cellular and antibody-mediated rejection in heart transplantation: In-depth pathologic and clinical observations. J Heart Lung Transpl (2016) 35(3):335–41. 10.1016/j.healun.2015.10.016 26586489

[25] Gupta SK Itagaki R Zheng X Batkai S Thum S Ahmad F. miR-21 promotes fibrosis in an acute cardiac allograft transplantation model. Cardiovasc Res (2016) 110(2):215–26. 10.1093/cvr/cvw030 26865549

[26] Hou LF He SJ Li X Yang Y He PL Zhou Y. Oral administration of artemisinin analog SM934 ameliorates lupus syndromes in MRL/lpr mice by inhibiting Th1 and Th17 cell responses. Arthritis Rheumatol (2011) 63(8):2445–55. 10.1002/art.30392 21484768

[27] Liao T Xue Y Zhao D Li S Liu M Chen J. In Vivo Attenuation of Antibody-Mediated Acute Renal Allograft Rejection by Ex Vivo TGF-beta-Induced CD4(+)Foxp3(+) Regulatory T Cells. Front Immunol (2017) 8:1334. 10.3389/fimmu.2017.01334 29085374 PMC5650643

[28] Roemhild A Otto NM Moll G Abou-El-Enein M Kaiser D Bold G. Regulatory T cells for minimising immune suppression in kidney transplantation: phase I/IIa clinical trial. BMJ (2020) 371:m3734. 10.1136/bmj.m3734 33087345 PMC7576328

[29] Hippen BE DeMattos A Cook WJ Kew CE,2 Gaston RS. Association of CD20+ infiltrates with poorer clinical outcomes in acute cellular rejection of renal allografts. Am J Transpl (2005) 5(9):2248–52. 10.1111/j.1600-6143.2005.01009.x 16095505

[30] Wu Y He S Bai B Zhang L Xue L Lin Z. Therapeutic effects of the artemisinin analog SM934 on lupus-prone MRL/lpr mice via inhibition of TLR-triggered B-cell activation and plasma cell formation. Cell Mol Immunol (2016) 13(3):379–90. 10.1038/cmi.2015.13 PMC4856803 25942599

[31] Abe T Ishii D Gorbacheva V Kohei N Tsuda H Tanaka T. Anti-huCD20 antibody therapy for antibody-mediated rejection of renal allografts in a mouse model. Am J Transpl (2015) 15(5):1192–204. 10.1111/ajt.13150 PMC5021301 25731734

[32] Djamali A Kaufman DB Ellis TM Zhong W Matas A Samaniego M. Diagnosis and management of antibody-mediated rejection: current status and novel approaches. Am J Transpl (2014) 14(2):255–71. 10.1111/ajt.12589 PMC4285166 24401076

[33] Gupta G Abu Jawdeh BG Racusen LC Bhasin B Arend LJ Trollinger B. Late antibody-mediated rejection in renal allografts: outcome after conventional and novel therapies. Transplantation (2014) 97(12):1240–6. 10.1097/01.TP.0000442503.85766.91 24937198

[34] Immenschuh S Zilian E Dammrich ME Schwarz A Gwinner W Becker JU. Indicators of treatment responsiveness to rituximab and plasmapheresis in antibody-mediated rejection after kidney transplantation. Transplantation (2015) 99(1):56–62. 10.1097/TP.0000000000000244 25121474

[35] Fabritius C Ritschl PV Resch T Roth M Ebner S Gunther J. Deletion of the activating NK cell receptor NKG2D accelerates rejection of cardiac allografts. Am J Transpl (2017) 17(12):3199–209. 10.1111/ajt.14467 PMC5694344 28805342

[36] Halloran PF Venner JM Madill-Thomsen KS Einecke G Parkes MD Hidalgo LG. Review: The transcripts associated with organ allograft rejection. Am J Transpl (2018) 18(4):785–95. 10.1111/ajt.14600 29178397

[37] Sun Q Zhang M Xie K Li X Zeng C Zhou M. Endothelial injury in transplant glomerulopathy is correlated with transcription factor T-bet expression. Kidney Int (2012) 82(3):321–9. 10.1038/ki.2012.112 22513824

[38] Li T Zhang Z Bartolacci JG Dwyer GK Liu Q Mathews LR. Graft IL-33 regulates infiltrating macrophages to protect against chronic rejection. J Clin Invest (2020) 130(10):5397–412. 10.1172/JCI133008 PMC7524467 32644975

[39] Dai H Lan P Zhao D Abou-Daya K Liu W Chen W. PIRs mediate innate myeloid cell memory to nonself MHC molecules. Science (2020) 368(6495):1122–7. 10.1126/science.aax4040 PMC7379379 32381589

